# Identification and Expression Analysis of Odorant-Binding and Chemosensory Protein Genes in Virus Vector *Nephotettix cincticeps*

**DOI:** 10.3390/insects13111024

**Published:** 2022-11-05

**Authors:** Xuefei Chang, Yaluan Bi, Haipeng Chi, Qi Fang, Zhaozhi Lu, Fang Wang, Gongyin Ye

**Affiliations:** 1 Shandong Engineering Research Center for Environment-Friendly Agricultural Pest Management, College of Plant Health and Medicine, Qingdao Agricultural University, Qingdao 266109, China; 2 State Key Laboratory of Rice Biology & Ministry of Agriculture and Rural Affairs Key Laboratory of Molecular Biology of Crop Diseases and Insects, Institute of Insect Sciences, Zhejiang University, Hangzhou 310058, China

**Keywords:** *Nephotettix cincticeps*, transcriptomic analyses, odorant binding proteins, chemosensory proteins, tissue expression profiles

## Abstract

**Simple Summary:**

*Nephotettix cincticeps*, one of the destructive pests of rice plants, not only directly damages hosts by sucking, but also indirectly transmits plant viruses in the field. However, the available resources of olfactory of *N. cincticeps* are little known, especially information regarding the odorant binding proteins (OBPs) and chemosensory proteins (CSPs) which are involved in the first barriers for receiving and sensing chemical signals. In the present study, the *N. cincticeps* adults head and the rest of the body transcriptome were sequenced and analyzed using Illumina sequencing. Twenty putative OBP and 13 CSP genes were identified. A total of 12 OBP and 10 CSP genes were detected, and nine OBP and three CSP genes were highly expressed in *N. cincticeps* antennae with tissue expression levels analysis. This study will help us understand the molecular mechanism of *N. cincticeps* in the detection and recognition of rice volatiles.

**Abstract:**

The insect odorant binding proteins (OBPs) and chemosensory proteins (CSPs) are involved in the perception and discrimination of insects to host odor cues. *Nephotettix cincticeps*, one of the destructive pests of rice plants, not only directly damages hosts by sucking, but also indirectly transmits plant viruses in the field. Previous study found that two rice volatiles ((*E*)-β-caryophyllene and 2-heptanol) induced by rice dwarf virus (RDV) mediated the olfactory behavior of *N. cincticeps*, which may promote virus dispersal. However, the OBPs and CSPs in *N. cincticeps* are still unknown. In this study, to identify the OBP and CSP genes in *N. cincticeps*, transcriptomic analyses were performed. In total, 46,623 unigenes were obtained. Twenty putative OBP and 13 CSP genes were discovered and identified. Phylogenetic analyses revealed that five putative OBPs belonged to the plus-C OBP family, and the other classic OBPs and CSPs were distributed among other orthologous groups. A total of 12 OBP and 10 CSP genes were detected, and nine OBP and three CSP genes were highly expressed in *N. cincticeps* antennae compared with other tissues. This study, for the first time, provides a valuable resource to well understand the molecular mechanism of *N. cincticeps* in the perception and discrimination of the two volatiles induced by RDV infection.

## 1. Introduction

Insect chemoreception system plays a pivotal role in many aspects of insect behaviors, such as foraging, mate recognition, oviposition, and avoiding predators [1,2]. In the insect olfactory perception process, various olfactory proteins containing odorant binding proteins (OBPs), chemosensory proteins (CSPs), odorant receptors (OR), ionotropic receptors (IR) and odorant degrading enzymes are involved [3,4]. Among these olfactory proteins, OBPs and CSPs could bind with fat-soluble odorant molecules to form complexes, the complexes are transferred to the olfactory receptors (ORs) through sensillar lymph, and then may activate the olfactory receptor neurons (ORNs) [3,5,6,7]. Actually, there is also evidence that OBPs are found in complex with pheromones, however, it is not sure if the complex OBP-pheromone is interacting with the ORs [8,9,10,11,12]. As the first barriers for receiving and sensing chemical signals, the OBPs and CSPs exert a crucial role in odor detection and recognition [13,14].

The insect OBPs are small water-soluble proteins, which are highly abundant in the sensillum lymph of insects [15,16]. Based on the number of conserved cysteine residues and their molecular structures, OBPs could be classed into several subfamilies including classic OBPs (six conserved cysteine residues, paired in three interlocked disulfide bridges), plus-C OBPs (two additional conserved cysteine and proline residues), minus-C OBPs (losing the second and fifth cysteines), dimer OBPs (two 6-cysteine signature motifs), and atypical OBPs (9–10 cysteines) [5,17]. Other physiological functions of OBPs other than acting as odor carriers have also been reported. For example, the NlugOBP3 is associated with the nymph survival of *Nilaparvata lugens* [18]. Some OBPs might be related to insect taste perception or involved in insecticide resistance [19,20,21,22,23,24]. CSPs are much smaller and more conserved than OBPs, which have the four typically conserved cysteine residues forming two disulphide bridges [6,25,26]. CSPs are present in insect various tissues. Other than perceiving host odor and sex pheromones [27,28], some CSP functions might be associated with limb repair, regulation of circadian cycles, growing development, immune responses, feeding behavior, and insecticide resistance [27,29,30,31,32,33,34].

The green rice leafhoppers (*Nephotettix cincticeps*) (Hemiptera: Cicadellidae) are one of the destructive pests of rice plants and distribute in the temperate regions of East Asia [35]. *N. cincticeps* not only directly produce damage by sucking sap from the phloem and xylem of host plants, but also indirectly affect the health of host plants. In fact, *N. cincticeps* transmit plant viruses such as the rice yellow stunt virus (RYSV) and rice dwarf virus (RDV) as an insect vector, which bring huge economic loss to rice plants [36,37]. Our previous study found that two rice volatiles ((*E*)-β-caryophyllene and 2-heptanol) induced by RDV infection influenced the non-viruliferous and viruliferous *N. cincticeps* olfactory behavior independently. (*E*)-β-caryophyllene acted as an attractant towards non-viruliferous *N. cincticeps*, mediating their preference for RDV-infected plants. In contrast, 2-heptanol acted as a repellent towards viruliferous *N. cincticeps*, leading them to prefer RDV-free plants [38]. Although the push-pull strategy formed by the action of two volatiles on non-viruliferous and viruliferous *N. cincticeps* would promote the spread of RDV, the molecular mechanism of *N. cincticeps* in the perception and discrimination of the two volatiles is still unknown. To well understand the above molecular mechanism, identification and annotation of the OBP and CSP genes in *N. cincticeps* are the first priority.

In this study, the *N. cincticeps* adults head and the rest of the body transcriptome were sequenced and analyzed using Illumina sequencing. The putative OBP and CSP genes in the *N. cincticeps* transcriptome were discovered and identified. To characterize these molecules, sequence alignment and phylogenetic analysis were investigated. The expression levels of OBP and CSP genes in different tissues of *N. cincticeps* were determined by quantitative real-time PCR (qRT-PCR). This study will give us a comprehensive characterization of OBPs and CSPs from *N. cincticeps* for the first time. Our findings will provide valuable insights into the design and implementation of novel strategies to control the damage caused by this rice pest.

## 2. Materials and Methods

### 2.1. Insects Rearing and Tissue Collection

The colony of *N. cincticeps* was obtained from the experimental rice fields of Zhejiang University, Hangzhou, China. These insects were reared on the susceptible rice “Taichung Native1” (TN1) seedlings covered with a nylon cage (80-mesh, 45 cm^3^) for four generations [39]. Approximately 600 newly emerged insects within 24 h were used for tissue collection. The different tissues of *N. cincticeps* including antennae, head, thoraxes, abdomen, leg and wing were quickly separated and stored in liquid nitrogen until RNA extraction. There were four biological replicates for different tissues.

### 2.2. cDNA Library Construction and Transcriptome Sequencing

Total RNA was independently extracted from the female, male head, and other body parts of adult *N. cincticeps* using Trizol reagent (Invitrogen, Waltham, CA, USA) following the manufacturer’s procedure. Quantity and purity of the RNA were analyzed with Bioanalyzer 2100 and RNA 1000 Nano LabChip Kit (Agilent, Santa Clara, CA, USA) (RIN number > 7.0). Poly(A) RNA is purified from total RNA using poly-T oligo-attached magnetic beads with two rounds of purification. After purification, the mRNA is fragmented into small pieces using divalent cations under elevated temperatures. Then the cleaved RNA fragments were reverse-transcribed to create the final cDNA library in accordance with the protocol for the mRNA-Seq sample preparation kit (Illumina, San Diego, CA, USA), the average insert size for the paired-end libraries was 300 bp (±50 bp). Then the paired-end sequencing on an IlluminaHiseq4000 at the (LC Sciences, Houston, TX, USA) was performed following the vendor’s recommended protocol.

### 2.3. De Novo Assembly, Unigene Annotation, and Functional Classification

Clean short reads were obtained by removing those containing an adapter or poly-N (>5%) and of low quality (Q ≤ 20) from the raw reads [40]. De novo assembly of the transcriptome was performed with Trinity 2.4.0 [41].

All assembled unigenes were aligned against the non-redundant (Nr) protein database (https://www.ncbi.nlm.nih.gov/ (accessed on 5 January 2021)), Gene ontology (GO) (http://geneontology.org/ (accessed on 5 January 2021)), SwissProt (http://www.expasy.ch/sprot/ (accessed on 5 January 2021)), Kyoto Encyclopedia of Genes and Genomes (KEGG) (http://www.genome.jp/kegg/ (accessed on 5 January 2021)) and eggNOG (http://eggnogdb.embl.de/ (accessed on 5 January 2021)) databases using DIAMOND [42] with a threshold of *E*-value < 0.00001.

### 2.4. Identification of OBP and CSP Genes

The head and other body transcriptome dataset of *N. cincticeps* was constructed in our laboratory using the Illumina platform. Putative NcinOBP and NcinCSP genes were identified by searching the keywords (odorant binding protein and chemosensory protein) in the annotated unigenes. In addition, we obtained the putative OBP and CSP genes from the genome dataset of *N. cincticeps* (provided by Professor Li Yi of Peking university). Then all the candidate OBP and CSP genes of *N. cincticeps* were confirmed by comparing the sequence against the NCBI non-redundant (nr) protein database using BLASTX. The conserved domains of these putative OBPs and CSPs were predicted by NCBI online tool (https://www.ncbi.nlm.nih.gov/Structure/cdd/wrpsb.cgi? (accessed on 28 January 2021). The open reading frame (ORF) of each candidate unigene was predicted using the ORF finder tool (https://www.ncbi.nlm.nih.gov/orffinder/ (accessed on 28 January 2021). The signal peptides of putative NcinOBP and NcinCSP proteins were predicted by the SignalP V 5.0 program (https://services.healthtech.dtu.dk/service.php?SignalP-5.0 (accessed on 28 January 2021).

### 2.5. Sequence Alignment and Phylogenetic Analysis

The amino acid sequences of candidate OBP and CSP of *N. cincticeps* (without signal peptide sequences) were aligned with their orthologs from other Hemipteran insect species by Clustal W [43]. A total of 161 OBP protein sequences from 9 Hemipteran species were used to construct the phylogenetic tree including 20 OBPs of *N. cincticeps* discovered in the present study, 44 OBPs of *Empoasca onukii*, 8 OBPs of *Bemisia tabaci*, 8 OBPs of *N. lugens*, 10 OBPs of *Sogatella furcifera*, 8 OBPs of *Laodelphax striatella*, 36 OBPs of *Apolygus lucorum*, 16 OBPs of *Adelphocoris lineolatus*, and 11 OBPs of *Acyrthosiphon pisum* (Sequences of OBPs are listed in Table S1). In addition, 103 CSP protein sequences from nine Hemipteran species were selected for the phylogenetic analysis including 13 CSPs of *N. cincticeps* identified in this study, 22 CSPs of *E. onukii*, 13 CSPs of *B. tabaci*, 10 CSPs of *N. lugens*, 9 CSPs of *S. furcifera*, 12 CSPs of *L. striatella*, 8 CSPs of *A. lucorum*, 8 CSPs of *A. lineolatus* and 8 CSPs of *Aphis gossypii* (Sequences of OBPs are listed in Table S2). These insect OBP and CSP sequences other than *N. cincticeps* were obtained from Zhao [44]. The OBP and CSP phylogenetic trees were constructed by MEGA 6.0 software (Mega Limited, Auckland, New Zealand) with the neighbor-joining method using the Poisson correction distance and 1000 bootstrap replications [45]. The final phylogenetic trees were optimized and visualized by an online tool, iTOL (https://itol.embl.de/ (accessed on 12 March 2021).

### 2.6. Tissue Expression Profile Analysis

The expression profiles for different tissues (female and male adults) of these candidate OBPs and CSPs were evaluated by qRT-PCR. The total RNA of different tissue of *N. cincticeps* including antennae, heads, thoraxes, abdomens, legs, and wings were extracted and reversely transcribed into cDNA as described previously [46]. The *β-actin* as a reference gene was used for normalizing target gene expression and to correct for sample-to-sample variation. The specific primers used for qRT-PCR analysis were designed with Primer 3.0 (http://bioinfo.ut.ee/primer3-0.4.0/ (accessed on 18 March 2021) (Table S3). cDNA templates and primer sets were mixed with SYBR^®^ Premix Ex Taq^TM^ II (Tli RNaseH Plus; Takara, Shiga, Japan) and real-time PCR was performed on the CFX Connect^TM^ Real-Time Detection System (Bio-Rad, Hercules, CA, USA). Negative controls were non-template reactions (replacing cDNA with sterile H_2_O). Four biological replicates were conducted for each experiment. The relative quantification was calculated using the comparative 2^−ΔΔCt^ method [47].

### 2.7. Statistical Analysis

All data were performed with SPSS 20.0 software(IBM, Armonk, NY, USA). The comparative analysis of each OBP and CSP genes among various tissues was determined using a one-way tested analysis of variance (ANOVA), followed by Tukey’s multiple range test (*p* < 0.05). The values were presented as the mean ± standard error.

## 3. Results

### 3.1. Overview of the Head and Rest of the Body Transcriptomes

Three mixed adult head and the rest of the body (10 females and 10 males) cDNA libraries were constructed and sequenced respectively using the IlluminaHiseq4000 platform. After filtering, a total of 55,049,478, 53,867,286, 54,310,996 (head samples) and 56,650,118, 55,478,556, 54,656,850 (rest of the body samples) clean data were generated with a Q20 scores (98.98%, 98.95%, 98.74% head samples; 98.89%, 98.98%, 98.50% rest of the body samples) (Table S4). Subsequently, all the clean reads were assembled together and obtained 46,623 unigenes with lengths ranging from 201 to 16,461 bp, with a mean length of 456 bp (Table S5).

### 3.2. Homology Searches and Functional Annotation

The database of NR, eggNOG, Swissprot, Pfam, KEGG, and GO were selected to annotate and verify the assembled sequences. Results showed that 16,495 (35.38%) unigenes were aligned in NR, 16,406 (35.19%) in eggNOG, 12,443 (26.69%) in Swissprot, 13,543 (29.05%) in Pfam, 12,142 (26.04%) in KEGG, and 13,891 (29.79%) in GO database respectively (Table S6).

All the unigenes were blasted against the nr sequence database in NCBI, with a threshold of *E*-value < 0.00001. Results suggested that the protein sequences of *N. cincticeps* were orthologs of proteins in *L. striatella* (13.34%) and *N. lugens* (13.10%) (Hemiptera), *Cimex lectularius* (4.73%) and *B. tabaci* (4.24%) (Hemiptera), and *Cryptotermes secundus* (9.54%) and *Zootermopsis nevadensis* (7.25%) (Isoptera) (Figure S1).

Only 13,891 (29.79%) assembled unigenes were annotated in three different functional groups (biological process, cellular component, and molecular function) with the GO category analysis (Figure S2). In the biological process category, the terms oxidation-reduction process and biological process were the most abundant. In the cellular component category, the cytoplasm and nucleus were most represented. In the molecular function category, the protein and ATP binding were the most abundant.

In KEGG annotation, 12,142 assembled transcripts were divided into six classes including organismal systems, metabolism, human diseases, genetic information processing, environmental information processing, and cellular process (Figure S3). In all pathways, several major ones in each class were identified containing transport and catabolism, signal transduction, translation, infectious diseases, carbohydrate metabolism, and the immune system.

### 3.3. Identification of Candidate OBP and CSP Genes in N. cincticeps

**Figure 1:**
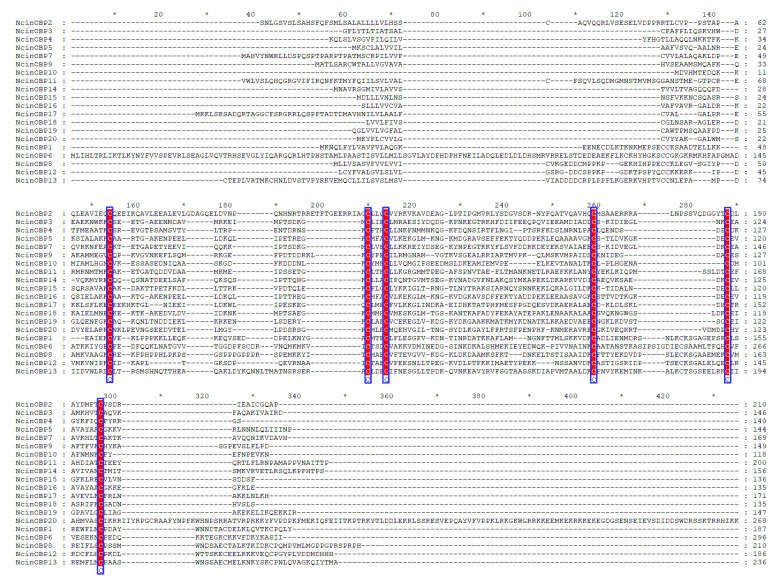
Sequence alignment of *N. cincticeps* OBPs. Sequences were aligned using the MEGA 6.0 and were presented using GeneDoc software (LynnonBiosoft, San Ramon, CA, USA). * indicates positions which have a single, fully conserved residue. Conserved amino acid residues are indicated by red letters. The six conserved Cys was indicated at the bottom of the alignment with a blue frame. The top 15 in order belonged to the “classic OBP family”, while sort the last five belonged to the “Plus-C OBP family”.

Based on the transcriptome and genome data, a total of 20 putative OBP genes were identified with the verification by BLASTX and BLASTN online tools (Table 1). The size of these OBP genes ranged from 118 to 296 amino acids. Most of the annotated OBP genes contained a signal peptide at their N-terminal part (other than NcinOBP4, 6, 11, 12, 15, and 17), which is a signature of secretory proteins. Based on the numbers and locations of the conserved cysteines, the candidate NcinOBP1, OBP6, OBP8, OBP12, and OBP13 genes belonged to the plus-C OBP family, while the other OBP genes were divided into the classic OBP family (Figure 1).

**Figure 2:**
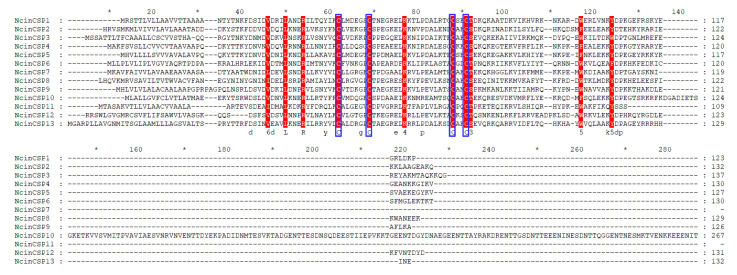
Sequence alignment of *N. cincticeps* CSPs. Sequences were aligned using the MEGA 6.0 and were presented using GeneDoc software. * indicates positions which have a single, fully conserved residue. Conserved amino acid residues are indicated by red letters. The four conserved Cys was indicated at the bottom of the alignment with a blue frame.

Similarly, 13 transcripts encoding putative CSPs were obtained in transcriptome and genome data (Table 2). Most of the CSP sequences contained the complete ORFs with the size ranging from 109 to 207 amino acids (except for CSP2, 8, 12), and all the sequences had a signal peptide. All the annotated CSP proteins had four conserved cysteines (Cys-X6-8-Cys-X18-19-Cys-X2-Cys) [48] (Figure 2).

### 3.4. Phylogenetic Analysis of N. cincticeps OBP and CSP Genes

**Figure 3:**
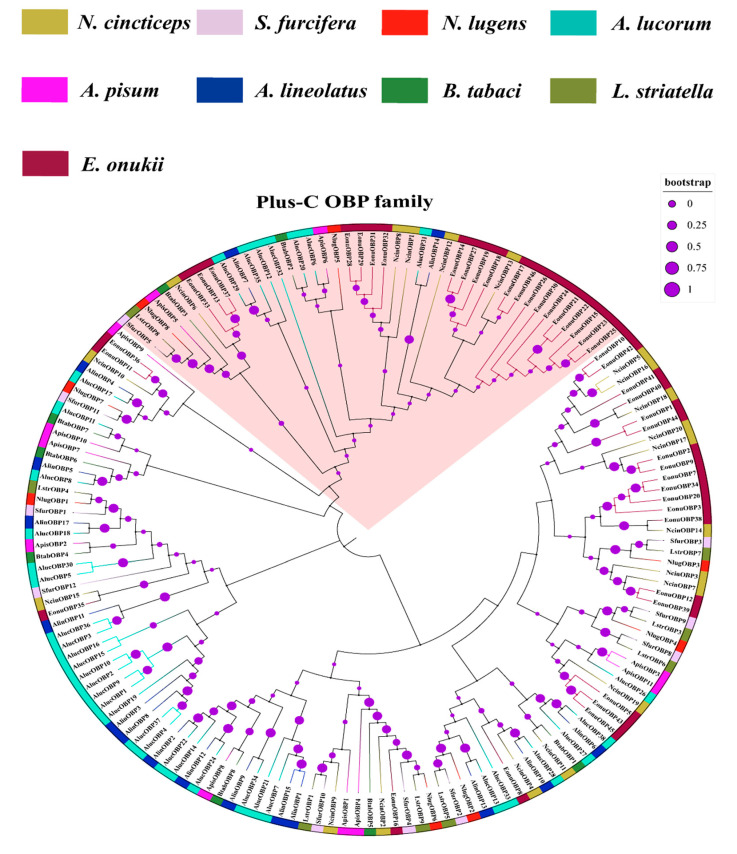
Neighbor-joining tree of 161 OBPs proteins from nine species of Hemiptera. Bootstrap values are shown. The Hemiptera species used to construct the tree are *E. onukii*, *N. lugens*, *S. furcifera*, *L. striatella*, *B. tabaci*, *A. lucorum*, *A. lineolatus*, *A. pisum* and *N. cincticeps*. The OBP phylogenetic trees were constructed by MEGA 6.0 software with the neighbor-joining method using the Poisson correction distance and 1000 bootstrap replications. The final phylogenetic trees were optimized and visualized by an online tool, iTOL (https://itol.embl.de/ (accessed on 12 March 2021)). The meaning of the pink highlight in the phylogenic tree was the “Plus-C” OBP family.

Due to *E. onukii* and *N. cincticeps* belonging to Cicadellidae, *L. striatella*, *S. furcifera*, *N. lugens*, and *N. cincticeps* having the same habitats, and the OBP/CSP of the other four insects being well annotated, thus we chose these species to construct the phylogenetic analysis. A phylogenetic tree containing of nine insect species of 161OBPs protein sequences in Hemiptera was constructed (Figure 3). These candidate OBP proteins were segmented into two classes of OBP sub-family (Plus-C and classic OBP family). NcinOBP1, OBP6, OBP8, OBP12, and OBP13 proteins were clustered into the Plus-C family, while the other OBP proteins were in the classic OBP family, indicating that these five genes have different evolutionary relationships with the other 15 OBP genes. The putative OBP proteins of *N. cincticeps* clustered into a different branch, but most sequences were gathered with the OBP proteins of *E. onukii*. In addition, NcinOBP4 was clustered with AlucOBP28 and AlinOBP10. NcinOBP9 was clustered with SfurOBP10 and LstrOBP1, while NcinOBP11 was clustered with BtabOBP1, implying that these genes may have similar functions.

**Figure 4:**
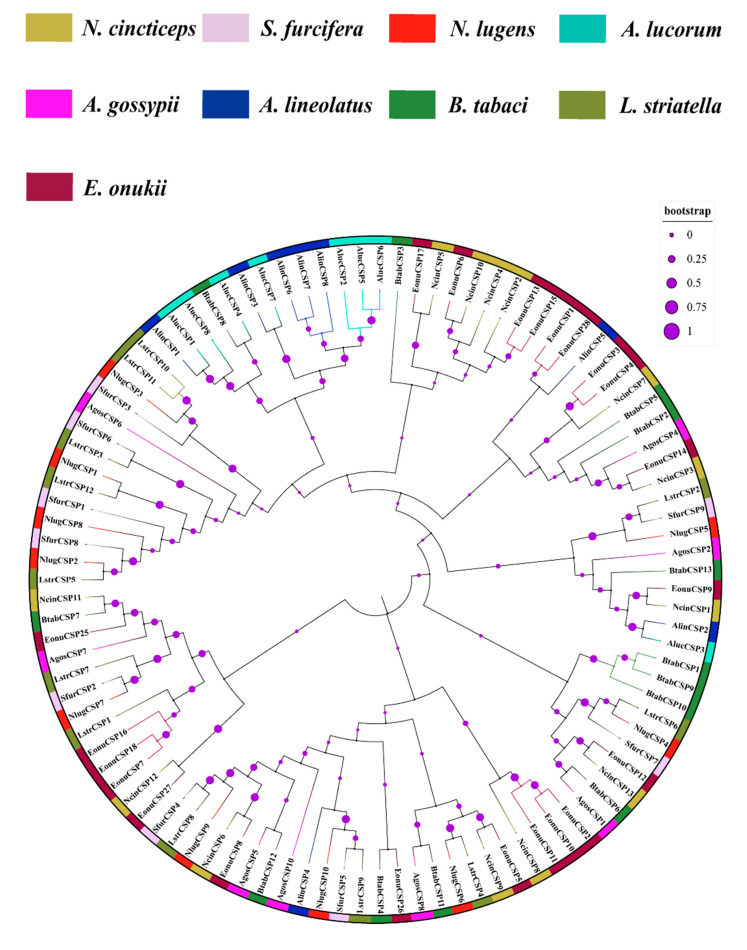
Neighbor-joining tree of 103 CSPs proteins from nine species of Hemiptera. Bootstrap values are shown. The Hemiptera species used to construct the tree are *E. onukii*, *N. lugens*, *S. furcifera*, *L. striatella*, *B. tabaci*, *A. lucorum*, *A. lineolatus*, *A. gossypii* and *N. cincticeps*. The OBP phylogenetic trees were constructed by MEGA 6.0 software with the neighbor-joining method using the Poisson correction distance and 1000 bootstrap replications. The final phylogenetic trees were optimized and visualized by an online tool, iTOL (https://itol.embl.de/ (accessed on 12 March 2021).

The neighbor-joining tree of 103 CSPs with nine insect species in Hemiptera was established (Figure 4). In this phylogenetic tree, all the putative CSPs could cluster with at least one hemipteran ortholog. Similarly, most of the NcinCSP proteins were clustered with the CSP proteins of *E. onukii*, because the two insects are members of Cicadellidae. Moreover, AgosCSP4 and NcinCSP3 were gathered with EonuCSP14, and NcinCSP11 was clustered with BtabCSP7, which suggests that these CSP genes maybe showed common functions.

### 3.5. Tissue Expression Levels of Candidate OBP and CSP Genes

**Figure 5:**
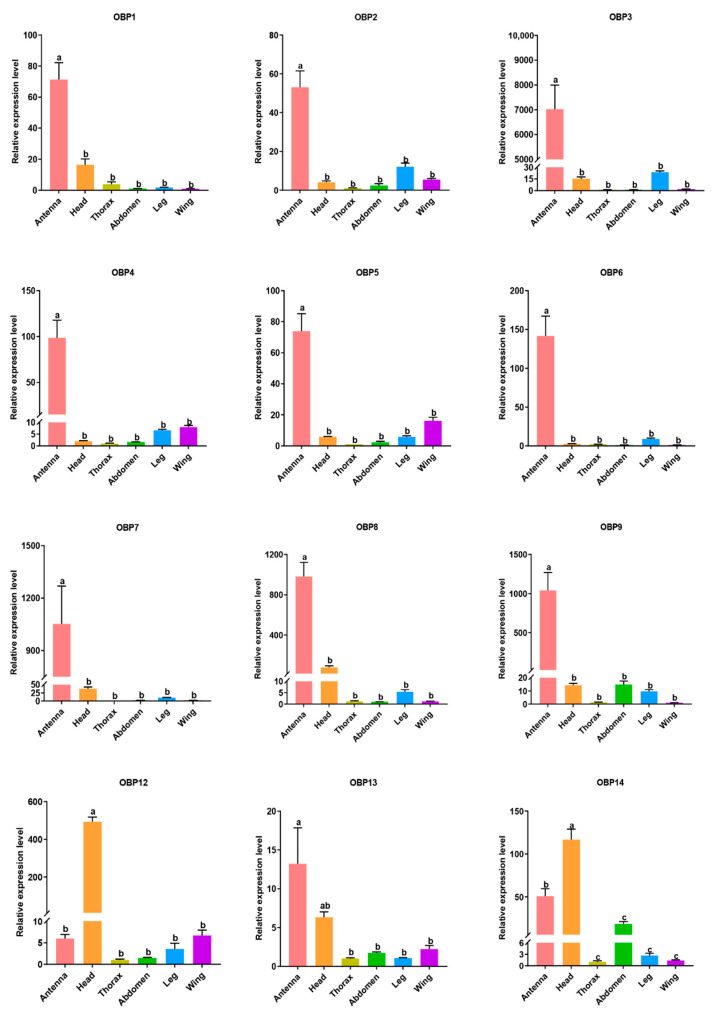
Relative expression levels of OBPs in different tissues of *N. cincticeps* analyzed by qRT-PCR. Four biological replicates were conducted for each experiment. The relative quantification was calculated using the comparative 2^−ΔΔCt^ method. The comparative analysis of each OBP genes among various tissues was determined using a one-way tested analysis of variance (ANOVA), followed by Tukey’s multiple range test (*p* < 0.05). Different lowercase letters indicate significant differences between different tissues. The values were presented as the mean ± standard error.

The expression levels of these identified OBP and CSP genes in the antennas, heads, thoraxes, abdomens, legs, and wings of *N. cincticeps* were measured by qRT-PCR. Nine candidate OBP genes (NcinOBP1–9) were mostly expressed in antennas, with 53 to 1042 times higher than in wings (Figure 5). While NcinOBP12 and NcinOBP14 exhibited a relative expression level of 493 and 116 times higher in heads than in wings respectively. The expression level of NcinOBP13 was higher in antennas and heads than in other tissues. The other eight putative OBP genes (NcinOBP10–11, 15–20) were not detected by qRT-RCR, maybe due to their expression levels being too low to detect.

**Figure 6:**
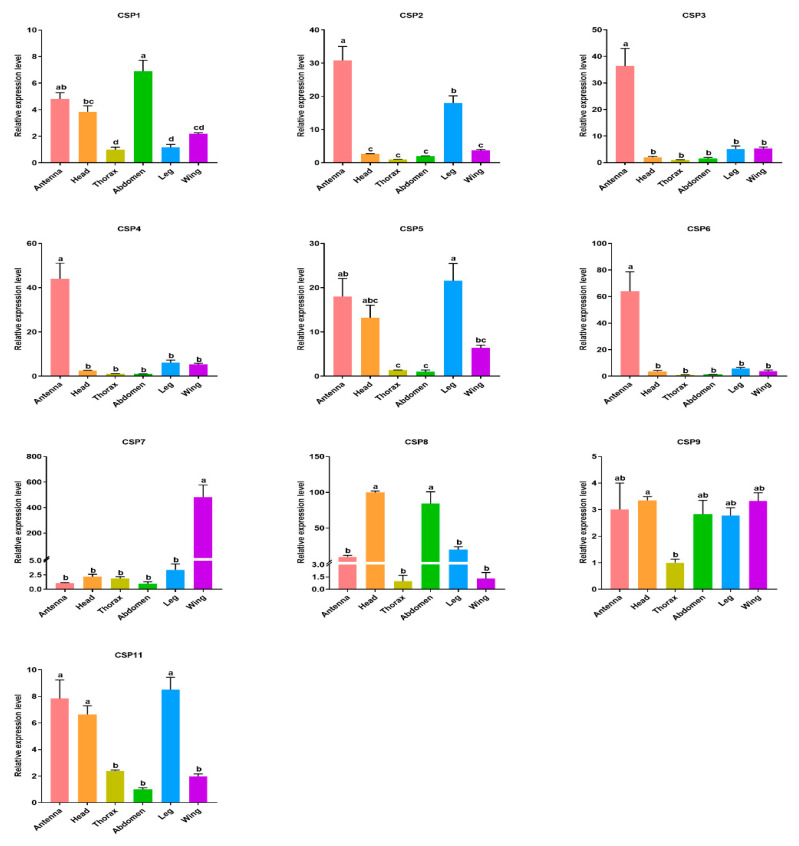
Relative expression levels of CSPs in different tissues of *N. cincticeps* analyzed by qRT-PCR. Four biological replicates were conducted for each experiment. The relative quantification was calculated using the comparative 2^−ΔΔCt^ method. The comparative analysis of each CSP genes among various tissues were determined using one-way tested analysis of variance (ANOVA), followed by Tukey’s multiple range test (*p* < 0.05). Different lowercase letters indicate significant differences between different tissues. The values were presented as the mean ± standard error.

For CSPs, four CSPs (NcinCSP2, 3, 4, 6) were specifically expressed in antennas, with 31 to 63 times higher than in wings (Figure 6). NcinCSP1 and NcinCSP5 were comparatively highly expressed in the abdomen and leg respectively, compared with the expression levels of other tissues. The expression level of NcinCSP7 was higher in the wing than that in other tissues. NcinCSP8 was comparatively highly expressed in the head and abdomen than that in other tissues, while NcinCSP11 was highly expressed in antennae, head, and leg than that in the thorax, abdomen, and wing. NcinCSP9 was more highly expressed in the head than that in the thorax. Due to the undetectable expression levels (Ct value > 35), the other three CSP genes (NcinCSP10, 12, 13) were not detected by qRT-RCR.

## 4. Discussion

*N. cincticeps* is one of the serious insect pests affecting rice production in East Asia [36]. RDV transmitted by *N. cincticeps* threatens rice crop yield and leads to enormous economic losses [49]. Two rice plant volatiles ((*E*)-β-caryophyllene and 2-heptanol) induced by RDV infection influenced the olfactory behavior of *N. cincticeps* independently [38]. The relevant OBP and CSP gene information will show a promising starting point to understand the molecular mechanism of *N. cincticeps* in the detection and recognition of the two volatiles. According to the transcriptomic analyses, 20 OBP genes and 13 CSP genes were identified. As well, nine of the OBP genes and three of the CSP genes were highly expressed in *N. cincticeps* antennae compared with other tissues. This information could be helpful for developing new prevention and control strategies with the two volatiles.

The number of OBP genes in *N. cincticeps* is more than in some other Hemipteran insect species. For examples, 15 OBP genes in *A pisum* [50], 8 OBP genes in *B. tabaci* [51], 8 OBP genes in *N. lugens* [52] and 16 OBP genes in *A. suturalis* [53] were reported. But it is significantly lower than in *E. onukii* (44 OBP genes) and in *A. lucorum* (38 OBP genes), respectively [44,54]. The number of *N. cincticeps* CSP genes, it is approximately close to *L. striatella* and *S. furcifera* [55], but it is markedly lower than in *E. onukii* (28 CSP genes) [44]. Different Hemipteran insect species have different numbers of OBP and CSP genes, which might be associated with insect habits or other environmental factors. For example, *E. onukii* had more OBP and CPS genes compared to other insects, which may be due to the wide range of host plants and complicated environment [56,57]. The internal mechanisms of different Hemipteran insect species with a different number of OBP and CSP genes need to be further investigated.

The evolutionary analysis will provide new insights into the evolution and function of *N. cincticeps* OBP and CSP genes with other Hemipteran species. In this study, most OBP and CSP genes of *N. cincticeps* were clustered into the OBP and CSP genes of *E. onukii*, indicating that the two insects had high homology relations. But the *N. cincticeps* OBPs are in different little branches, and the OBP sequences consistency is low, implying that the functions of these OBPs are differentiation. The OBP genes were divided into two classes of OBP sub-family (Plus-C and classic OBP family) (Figure 3). The plus-C NcinOBP genes were subsequently clustered into two independent orthologous groups. NcinOBP6 was in one orthologous group, and NcinOBP1, OBP8, OBP12, and OBP13 were in another orthologous group, suggesting that the two plus-C OBP genes orthologous groups were perhaps derived from common ancestors and then diverged before speciation [58]. The NcinCSP genes were clustered with different hemipteran ortholog groups, indicating that the CSP genes have an earlier origin and are more conserved compared with the OBP genes [59,60]. *L. striatella*, *S. furcifera*, *N. lugens,* and *N. cincticeps* have the same habitats (feeding host plants of Poaceae, such as rice and maize). Previous studies showed that (*E*)-β-caryophyllene had attractions to *S. furcifera* and *N. cincticeps* [38,61]. The phylogenetic tree suggested that NcinOBP9 was clustered with SfurOBP10. Thus, we speculated that the two OBP genes might have similar functions.

Different locations of OBP and CSP genes in insect tissues might have different functions [62,63,64,65,66]. Therefore, investigation of the tissue expression patterns of OBP and CSP genes in *N. cincticeps* might help to predict their physiological functions. Herein, we found that most OBP genes (9 of 12) were significantly expressed in *N. cincticeps* antennae compared with other tissues, which is consistent with other Hemipteran species (e.g., [55,59,66]). Insect antennae are thought to be a mainly olfactory organs to perceive the host plant volatiles, alarm pheromones, and sex pheromones [67,68,69,70]. Thus, we speculate that the nine OBPs and three CSPs, which are highly expressed in *N. cincticeps* antennae, might be involved in the perception and discrimination of the two rice volatiles induced by RDV infection. In addition, two OBP (NcinOBP12, OBP14) and two CSP genes (NcinCSP1, CSP5, CSP7, CSP8) are markedly expressed in *N. cincticeps* head and leg compared with antennae respectively, which may play other functions, for instance, carriers of visual pigments, regeneration and development, anti-inflammatory action, nutrition or insecticide resistance [4,24,71]. The specific functions of these OBP and CSP genes will be studied in the next step.

## 5. Conclusions

In summary, a total of 20 putative OBP and 13 CSP genes are identified from the *N. cincticeps* transcriptome. Twelve OBP and 10 CSP genes are detected by qRT-PCR and, nine OBP and three CSP genes are highly expressed in *N. cincticeps* antennae compared with other tissues. Further study needs to analyze the effects of RDV infection on the expression of these OBP and CSP genes, which could be selected as the potential target proteins. Then the molecular mechanism of non-viruliferous and viruliferous *N. cincticeps* perception of the two volatiles induced by RDV would be analyzed respectively, which will provide a theoretical basis and technical support for the ecological prevention and control of plant diseases transmitted by insect vectors.

## Figures and Tables

**Table 1:** List of identified OBP genes of *N. cincticeps*.

Gene ID	Gene Name	ORF	SP		Best Blastx Match				
		(aa)	(aa)	Species	Gene Name	Acc. No	Score	*E*-Value	Identity (%)
evm.model.scaffold644.6	OBP1	187	17	*Empoasca onukii*	OBP6	AWC68015.1	97.6	8 × 10^−22^	28.26
TRINITY_DN14345_c0_g1	OBP2	210	33	*Empoasca onukii*	OBP16	AWC67989.1	258	1 × 10^−84^	64.43
TRINITY_DN20712_c0_g1	OBP3	146	16	*Empoasca onukii*	OBP12	AWC67992.1	145	1 × 10^−41^	50.41
TRINITY_DN17302_c1_g2	OBP4	140	No	*Empoasca onukii*	OBP8	AWC67990.1	125	6 × 10^−34^	42.96
TRINITY_DN20407_c2_g4	OBP5	144	19	*Empoasca onukii*	OBP42	AWC67997.1	165	2 × 10^−49^	61.54
evm.model.scaffold232.48	OBP6	296	No	*Empoasca onukii*	OBP13	AWC68014.1	286	3 × 10^−93^	60.73
evm.model.scaffold64.23	OBP7	169	44	*Empoasca onukii*	OBP12	AWC67992.1	176	2 × 10^−53^	58.57
TRINITY_DN19892_c0_g6	OBP8	210	19	*Empoasca onukii*	OBP22	AWC68011.1	165	8 × 10^−48^	46.46
evm.model.scaffold64.19	OBP9	149	24	*Subpsaltria yangi*	OBP2	AXY87861.1	125	7 × 10^−34^	42.22
evm.model.scaffold94.54	OBP10	118	26	*Empoasca onukii*	OBP11	AWC67987.1	124	1 × 10^−33^	49.55
TRINITY_DN16837_c1_g7	OBP11	200	No	*Ctenocephalides felis*	GOBP 19d-like	XP_026482103.1	62.0	2 × 10^−8^	30.91
TRINITY_DN16837_c1_g7	OBP12	236	No	*Empoasca onukii*	OBP22	AWC68011.1	165	8 × 10^−48^	46.46
TRINITY_DN16837_c1_g7	OBP13	143	21	*Empoasca onukii*	OBP46	AWC68013.1	121	7 × 10^−31^	36.02
evm.model.scaffold64.25	OBP14	156	23	*Empoasca onukii*	OBP38	AWC68002.1	180	4 × 10^−55^	64.12
evm.model.scaffold371.21	OBP15	135	No	*Empoasca onukii*	OBP49	AWC68018.1	99.0	2 × 10^−13^	38.28
TRINITY_DN21214_c0_g2	OBP16	135	17	*Empoasca onukii*	OBP2	AWC67994.1	188	3 × 10^−58^	72.95
TRINITY_DN20748_c1_g13	OBP17	171	No	*Empoasca onukii*	OBP42	AWC67997.1	159	2 × 10^−47^	58.96
TRINITY_DN2151_c0_g1	OBP18	135	16	*Empoasca onukii*	OBP42	AWC67997.1	112	1 × 10^−28^	45.97
TRINITY_DN17208_c0_g2	OBP19	147	20	*Empoasca onukii*	OBP5	AWC67993.1	84.3	1 × 10^−17^	35.00
TRINITY_DN20849_c1_g2	OBP20	267	17	*Subpsaltria yangi*	OBP1	AXY87860.1	59.3	5 × 10^−7^	27.97

**Table 2:** List of identified CSP genes of *N. cincticeps*.

Gene ID	Gene Name	ORF	SP		Best Blastx Match				
		(aa)	(aa)	Species	Gene Name	Acc. No	Score	*E*-Value	Identity (%)
TRINITY_DN19371_c0_g5	CSP1	123	18	*Empoasca onukii*	CSP9	AWC68028.1	202	1 × 10^−64^	77.69
TRINITY_DN20809_c0_g2	CSP2	128	17	*Empoasca onukii*	CSP13	AWC68032.1	143	2 × 10^−41^	57.89
TRINITY_DN19425_c1_g3	CSP3	137	24	*Empoasca onukii*	CSP14	AWC68033.1	166	4 × 10^−50^	60.47
TRINITY_DN18965_c0_g5	CSP4	130	19	*Nilaparvata lugens*	putative CSP8	ACJ64054.1	152	6 × 10^−45^	52.31
TRINITY_DN20331_c0_g1	CSP5	132	21	*Empoasca onukii*	CSP17	AWC68036.1	166	3 × 10^−50^	72.22
TRINITY_DN18304_c1_g6	CSP6	130	15	*Empoasca onukii*	CSP8	AWC68026.1	194	8 × 10^−61^	67.97
evm.model.scaffold1081.12	CSP7	119	19	*Empoasca onukii*	CSP4	AWC68023.1	147	3 × 10^−43^	56.90
TRINITY_DN21169_c0_g11	CSP8	124	18	*Empoasca onukii*	CSP2	AWC68021.1	124	7 × 10^−34^	47.93
TRINITY_DN16691_c0_g1	CSP9	126	15	*Empoasca onukii*	CSP5	AWC68022.1	167	7 × 10^−71^	67.77
TRINITY_DN15719_c0_g3	CSP10	267	17	*Nezara viridula*	CSP6	AWC68037.1	158	4 × 10^−45^	59.13
TRINITY_DN13577_c0_g1	CSP11	109	20	*Lygus hesperus*	putative CSP1	APB88037.1	161	6 × 10^−9^	68.18
TRINITY_DN20737_c0_g2	CSP12	123	18	*Empoasca onukii*	CSP27	AWC68029.1	178	4 × 10^−55^	83.84
evm.model.scaffold312.53	CSP13	127	17	*Empoasca onukii*	CSP13	AWC68032.1	162	1 × 10^−48^	64.41

## Data Availability

The datasets presented in this study can be found in online repositories. The names of the repository/repositories and accession number(s) can be found below: https://www.ncbi.nlm.nih.gov/sra/PRJNA869491 (accessed on 22 August 2022).

## Supplementary Materials

The following supporting information can be downloaded at: https://www.mdpi.com/article/10.3390/insects13111024/s1. Table S1. OBP sequences used to construct the phylogenetic tree. Table S2. CSP sequences used to construct the phylogenetic tree. Table S3. Primers used for qRT-PCR analysis of OBP and CSP genes of *N. cincticeps*. Table S4. Overall profile analysis of sequencing data. Table S5. The summary of distribution of assembled length in *N. cincticeps* transcriptome. Table S6. The number of unigenes annotated in different database. Figure S1. Species distribution of top best hits of the BLASTx results. Figure S2. Gene Ontology (GO) classification of the 46,623 unigenes of *N. cincticeps*. Figure S3. KEGG classification of the 46,623 unigenes *N. cincticeps*.
