# Supplementary material for: Identification and Expression Analysis of Odorant-Binding and Chemosensory Protein Genes in Virus Vector Nephotettix cincticeps
Source: Insects. 2022 Nov 5;13(11):1024. doi: 10.3390/insects13111024 (PMC9698027; doi:10.3390/insects13111024)
Supplement: Supplementary file 1 [file insects-13-01024-s001.zip › Supporting Information.pdf]

Identification and expression analysis of odorant-binding and chemosensory protein genes in *Nephotettix cincticeps*, a main vector of rice dwarf virus in Asia

Xuefei Chang[1,2], Haipeng Chi[1], Yaluan Bi[2], Qi Fang[2], Zhaozhi Lu[1], Fang Wang[2]*, Gongyin Ye[2]*

[1]Shandong Engineering Research Center for Environment-Friendly Agricultural Pest Management, College of Plant Health and Medicine, Qingdao Agricultural University, Qingdao 266109, China

[2]State Key Laboratory of Rice Biology & Ministry of Agriculture and Rural Affairs Key Laboratory of Molecular Biology of Crop Diseases and Insects, Institute of Insect Sciences, Zhejiang University, Hangzhou 310058, China

***Correspondence:**

Fang Wang, wangf121@zju.edu.cn

Gongyin Ye, email: chu@zju.edu.cn.

Supporting Information

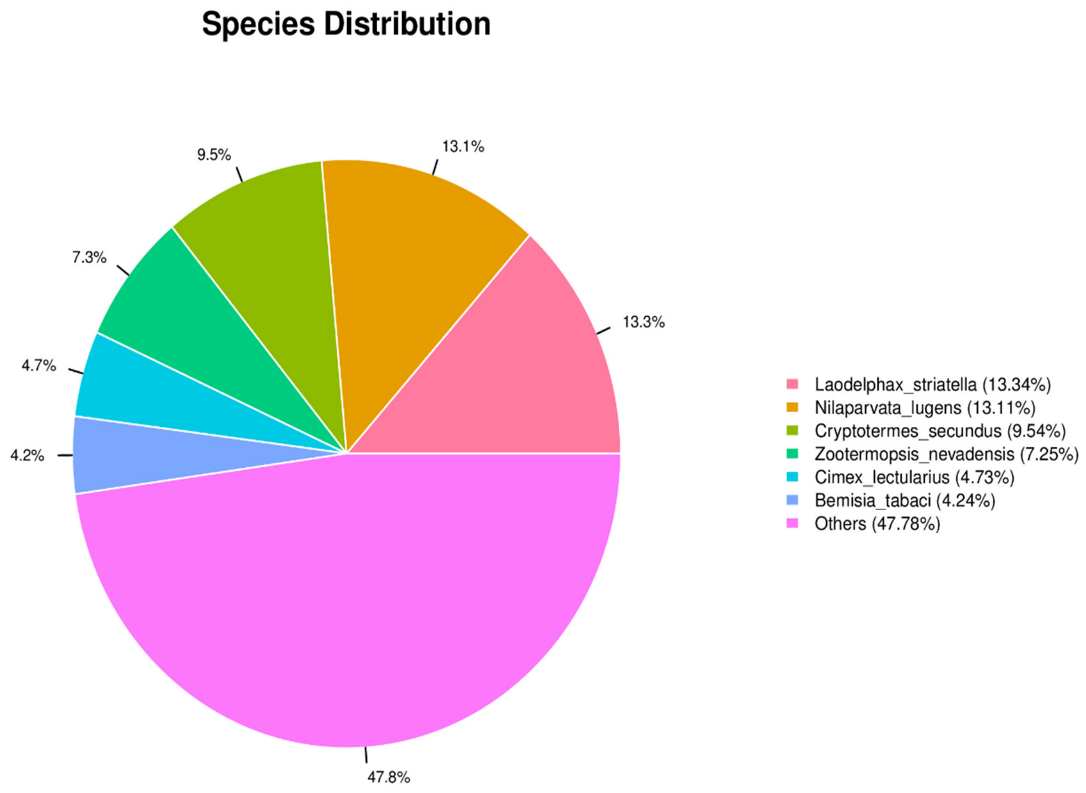

Figure S1 Species distribution of top best hits of the BLASTx results.

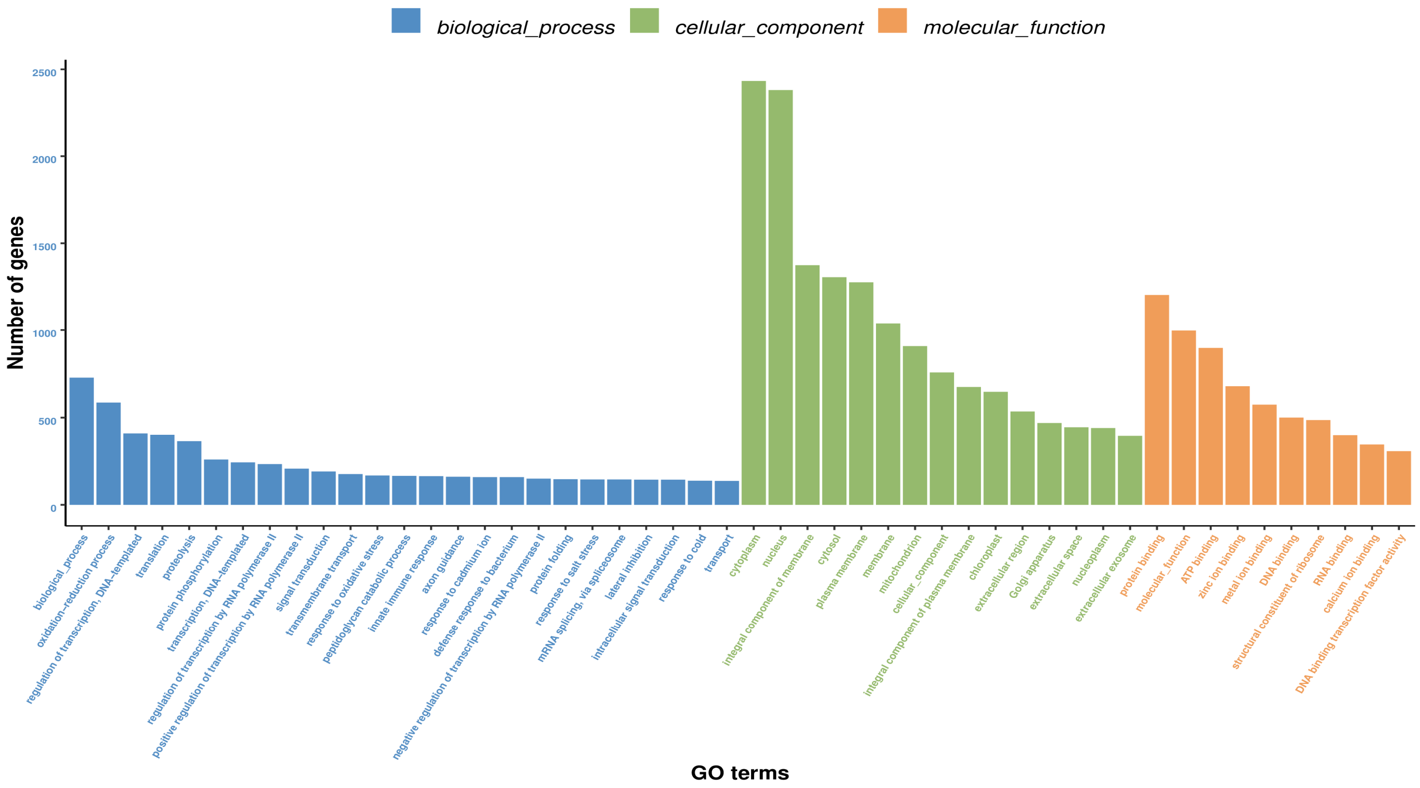

Figure S2 Gene Ontology (GO) classification of the 46,623 unigenes of *N. cincticeps*.

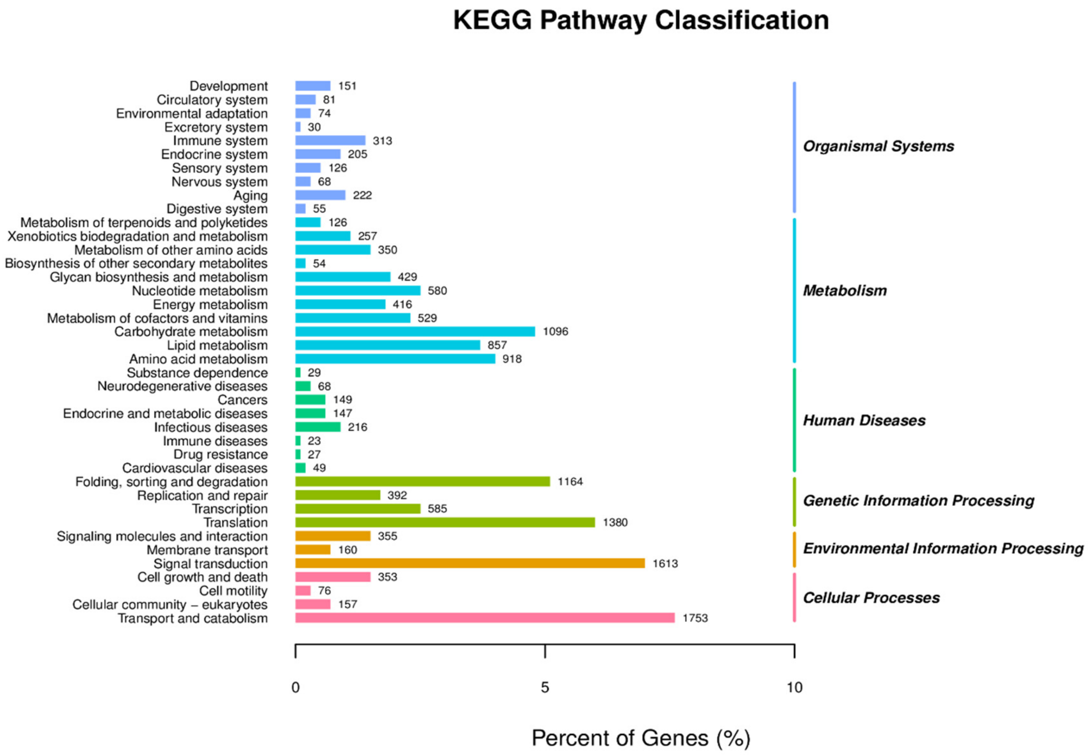

Figure S3 KEGG classification of the 46,623 unigenes *N. cincticeps*.
